# A Systematic Approach for Stronger Documentation of Anterior Cruciate Ligament Graft Choice

**DOI:** 10.7759/cureus.19017

**Published:** 2021-10-25

**Authors:** Christina Arida, Dimitrios S Mastrokalos, Andreas Panagopoulos, John Vlamis, Ioannis K Triantafyllopoulos

**Affiliations:** 1 3rd Orthopaedic Department, KAT Hospital, National and Kapodistrian University of Athens Medical School, Athens, GRC; 2 1st Orthopaedic Department, Attikon Hospital, National and Kapodistrian University of Athens Medical School, Athens, GRC; 3 Orthopaedic Department, University of Patras Medical School, Patras, GRC; 4 Orthopaedics, HYGEIA Private Hospital, Athens, GRC

**Keywords:** rupture, knee osteoarthritis, bone-patellar tendon-bone grafts, hamstring autograft, anterior cruciate ligament (acl)

## Abstract

Numerous studies have focused on determining the optimal choice between the two most used anterior cruciate ligament (ACL) reconstruction autografts. In order to address this matter, we performed a systematic review of every meta-analysis published on the PubMed platform between 2001 and 2020, comparing the functional outcomes, the static stability parameters, as well as the postoperative and long-term complications of the patellar tendon (BPTB) autograft and hamstrings (HT). We retrieved a total of 26 meta-analyses that met our criteria, and the characteristics and outcomes of every meta-analysis, as well as subgroup analysis regarding the type of the study design, number of strands of HT autograft, and fixation method, were extensively recorded. The majority of the meta-analyses showed that there were no significant differences between BPTB and HT in terms of functional outcomes and static stability parameters while HT autografts seem to be superior to BPTB regarding kneeling pain and anterior knee pain. Other outcomes seem to be affected by the number of strands of the HT autograft, the fixation technique, and the type of study design, indicating superiority of the four-strand HT autograft with the use of an extra-cortical button fixation. Overall, there is no clear superiority of BPTB over HT autografts for ACL reconstruction, as both types present similar outcomes in the majority of postoperative parameters. Autograft selection should be individualized according to each patient’s needs and more RCTs are warranted in order to reach safer results on the appropriate autograft type.

## Introduction and background

As a critical component of knee anterior-posterior and rotational stability, the anterior cruciate ligament (ACL) and its bony attachments have been investigated for more than 30 years and the findings have resulted in modifications in the techniques of reconstruction following rupture [1]. In the ACL reconstruction technique, several debates have engaged orthopedic surgeons and researchers, and the efficacy of ACL reconstruction is mainly attributed to the type of graft [2]. The goals of ACL reconstruction are the restoration of normal knee anatomy and function, re-establishment of biological and biomechanical knee homeostasis, and prevention of osteoarthritis (OA) [3]. Autografts are the preferred options due to reduced foreign body rejection, potential allergic reactions, and any disease transmission, and the most common choices are bone-patellar-tendon-bone (BPTB) and hamstring tendon (HT, semitendinosus, and gracilis). Despite the advantages that autografts have, each type of autograft may have specific complications, mainly related to the harvesting site, and results in short-term, medium-term, or long-term clinical effects. An ideal graft has not yet been reported in the available literature [4], in spite of the fact that strong proponents for each graft type exist, and certain advantages and disadvantages have been suggested. The purpose of this study was to conduct a systematic review of the literature comparing BPTB autograft versus HT autograft for ACL reconstruction and present every meta-analysis that has been recorded till today, comparing these two autografts, in an attempt to provide guidelines and enhance the critical thinking approach on the controversy regarding ACL reconstruction.

## Review

Material and methods

In June 2021, we searched the PubMed platform for meta-analyses, published between January 1, 2001, and December 31, 2020, directly comparing the BPTB and HT grafts, on ACL reconstruction using patellar tendon or hamstrings tendon. To identify outcome studies on ACL reconstruction, we used certain keywords: anterior cruciate ligament, reconstruction, patellar tendon, hamstrings, BPTB, semitendinosus, gracilis, autograft in multiple combinations, and a more detailed search strategy (ACL OR anterior cruciate ligament), (patella OR patellar OR bone patellar tendon OR bone patella tendon), (hamstring OR semitendinosus OR gracilis tendon). Additional literature was identified by searching the reference lists of meta-analyses to ensure that all relevant meta-analyses would be included in this systematic review. The objectives, analysis methods, and inclusion and exclusion criteria were determined after the data were collected and evaluated.

Inclusion and exclusion criteria

The criteria were as follows: 1. The language of the included studies was restricted to English; 2. Both abstract and full text had to be available online; 3. Any meta-analysis comparing additional types of grafts was excluded; 4. The number of strands of the autografts and the fixation in the proximal and distal parts were not considered limitations; 5. Systematic reviews that did not perform a meta-analysis were excluded. 6. Cadaveric, animal, or other laboratory studies were also excluded.

Data extraction

The following data were obtained from the selected meta-analyses: 1. Author and year of the meta-analysis; 2. Number and type of studies, number of participants, minimum and maximum follow-up periods, and number of strands of the HT graft used in each meta-analysis; 3. Functional outcomes, such as the International Knee Documentation Committee (IKDC) score, return to pre-injury activity level (RIAL), Lysholm and Tegner knee score, Cincinnati Knee Rating System, single-leg hop test, extension strength, flexion strength; 4. Static stability parameters, such as side-to-side difference, Lachman test, pivot-shift test; 5. Postoperative complications, such as kneeling pain (KP), anterior knee pain (AKP), extension loss, flexion loss, infection rate, graft failure, and contralateral ACL rupture (CACLR); 6. Long-term complications, such as osteoarthritis (OA).

The results of each meta-analysis were categorized as a non-significant difference between the two groups if the p-value was reported to be >0.05 and in favor of the BPTB group or HT group if the p-value was reported to be ≤ 0.05. If authors reported a trend to favor one graft over another, the p-value was also recorded.

Results

Study Characteristics

Our search strategy resulted in a total of 27 meta-analyses that met inclusion and exclusion criteria [5-30]. One of them by Shi and Yao 2011 [31] was excluded since it wasn’t available online. The number of primary studies included in these meta-analyses ranged from four to 69 and included both randomized controlled trials (RCTs) and observational studies.

Of a total of 26 meta-analyses, 11 were limited to only three or four-strand HT [7-9,13,20-21,23-24,26,28-29], eight did not specify [5-6,12,14,17,19,22,27], and the other seven used multi strands [10-11,15-16,18,25,30]. Six meta-analyses included only RCTs in their analysis [13,18,23,26,28-29] and five of them compared a BPTB graft to onlya four-strand HT graft [13,23,26,28-29].

Sixteen of the studies reported the sample size of patients undergoing ACL rupture (ACLR) with a BPTB autograft, as well as the sample size of patients with an HT autograft [5-8,10-11,13,18,21-22,25-30]. The remaining five reported a total number of patients [15-16,20,23-24], one reported the total sample and specified the graft the majority received, but a small number remained unknown [19]. Another one reported a number of series [9] and three did not specify the sample size [12,14,17].

Twenty-one of the studies reported on short-term outcomes [5-13,15-19,21-25,27,30], four on long-term outcomes [14,20,28-29], and another reported one on all three, short-, mid-, and long-term outcomes [26].

All meta-analyses included male and female patients, except from Tan et al. who focused on the female population only [25].

The characteristics of each included meta-analysis are shown in Table 1.

**Table 1:** Characteristics of each meta-analysis RCT: randomized controlled trial, PCS: prospective cohort studies

Meta-analysis	No. and type of studies used in the metanalysis	no. of patients	Min / max follow up	no. HT strands
Yunes 2001 [5]	4 controlled studies	234 BPTB 190 HT	2 years/-	Not reported
Freedman 2003 [6]	21 BPTB studies 13 HT studies	1348 BPTB 628 HT	24months/100 months or NA	Not reported
Forster 2005 [7]	6 RCTs or quasi- RCTs	235 BPTB 240 HT	2 years/-	4
Goldblatt 2005 [8]	11 prospective studies (random or sequentially)	515 BPTB 524 HT	2 years/-	3-4
Prodromos 2005 [9]	64 published reports of ACLR series	32 BPTB series 32 HS series	2 years/-	2 and 4 separately evaluated
Biau 2006 [10]	18 RCTs or quasi- RCTs	765 BPTB 747 HT	12 months/ 102 months	Multi (2 NR, 4 2HT, 4 3-4HT, 10 4HT)
Biau 2007 [11]	7 RCTs and 7 quasi- RCTs	649 BPTB 614 HT	24 months/ 102 months	2-5 (3 2HT, 2 3-4HT, 8 4HT, 1 5HT)
Poolman 2007 [12]	14 RCTs or quasi- RCTs (pooled by Biau 2006)	Not reported	Not reported	Not reported
Biau 2009 [13]	6 RCTs	216 BPTB 207 HT	24 months /-	4
Magnussen 2010 [14]	5 RCTs, 2 PCT	Not reported	5 years/ 8.5 years	Not reported
Mohtadi 2011 [15]	8 RCTs or 11 quasi- RCTs	1597 total	24 months/ 102 months	2-5
Li 2011 [16]	8 RCTs and 11 quasi- RCTs	1643 total	12 months/ 102 months	2-5 (3 2HT, 2 3-4HT, 13 4HT, 1 5HT)
Xergia 2011 [17]	4 RCTs and 3 non-RCTs	Not reported	Only 12 months evaluation	Not reported
Li 2012 [18]	9 RCTs	341 BPTB 397 HT	24months/ 105 months	2-4 (2 2HT, 2 3-4HT, 5 4HT)
Ardern 2014 [19]	69 articles	7556 (4405 BPTB, 2427 HT, 335 NA)	12 months/ 156 months	Not reported
Xie 2014 [20]	12 RCTs and 2 PCS	1443 total	5 years/ 15 years	4-5 (13 4HT, 1 5HT)
Xie 2015 [21]	14 RCTs and 8 PCS	931 BPTB 999 HT	24 months/ 180 months	4
Gabler 2016 [22]	28 RCTs or PCS	375 BPTB 2032 HT	24 months/ 132 months	Not reported
Chee 2016 [23]	19 RCTs	1784 total	24 months/ 132 months	4
Samuelsen 2017 [24]	14 RCTs, 10 PCS and one high-quality national registry study	47613 total	24 months/ 240 months	4
Tan 2018 [25]	10 PCS	278 BPTB 339 HT	9 months/ 240 months	multi
He 2019 [26]	23 RCTs	993 BPTB 993 HT	short-term ( ≤2 years) (12 trials)	4
			mid-term (3-5 years) (4 trials)	
			long-term ( > 5 years) (7 trials)	
Chen 2019 [27]	5 RCTs and PCS	233 BPTB 248 HT	24 months/ 182 months	Not reported
Zhao 2020 [28]	15 RCTs	610 BPTB 688 HT	60 months/ 204 months	3 3-4HT,11 4HT, 1 5HT
Chen 2020 [29]	9 RCTs	313 BPTB 317 HT	5 years/ 17 years	4
Zhou 2020 [30]	11 RCTs, 3 prospective comparative studies, 1 national registry	1760 BPTB 3801 HT	2 years/ 20 years	multi

Outcomes

The outcomes of each included meta-analysis are shown in Tables 2-4.

**Table 2:** Functional outcomes RIAL: return to preinjury activity level, NS: nonsignificant difference, Ext.strength: extension strength, Flex.strength: flexion strength HT: in favor of hamstrings autograft, BPTB: in favor of bone patella tendon bone autograft

Meta-analysis	IKDC	Lysholm	RIAL	Tegner	Cincinnatti	Single-leg hop test	Ext.strength	Flex.strength
Yunes 2001 [5]	-	-	BPTB	-	-	-	-	-
Freedman 2003 [6]	Data not combined	Data not combined	NS	Data not combined	-	-	-	-
Forster 2005 [7]	Data not combined	Data not combined	NS	-	NS	-	60 °NS	60° BPTB
Goldblatt 2005 [8]	NS	Only graphical analysis with no apparent differences	NS	Only graphical analysis with no apparent differences	-	-	Not possible because of the wide variability in reporting among studies	Not possible because of the wide variability in reporting among studies
Prodromos 2005 [9]	-	-	-	-	-	-	-	-
Biau 2006 [10]	-	-	-	-	-	-	-	-
Biau 2007 [11]	NS	-	NS	-	-	-	-	-
Poolman 2007 [12]	-	-	-	-	-	-	-	-
Biau 2009 [13]	-	-	-	-	-	-	-	-
Magnussen 2010 [14]	-	-	-	-	-	-	-	-
Mohtadi 2011 [15]	NS	NS	NS	NS	NS	NS	60° NS 180° NS	60° BPTB 180° BPTB
Li 2011 [16]	-	-	-	-	-	-	-	-
Xergia 2011 [17]	-	-	-	-	-	-	60° HT 180° HT	60° BPTB 180° BPTB
Li 2012 [18]	NS	-	-	-	-	-	-	-
Ardern 2014 [19]	-	-	BPTB	-	-	-	-	-
Xie 2014 [20]	NS	-	NS	-	-	-	-	-
Xie 2015 [21]	NS	NS	BPTB	-	-	-	-	-
Gabler 2016 [22]	-	-	-	-	-	-	-	-
Chee 2016 [23]	NS	NS	NS	NS	-	-	-	-
Samuelsen 2017 [24]	-	-	-	-	-	-	-	-
Tan 2018 [25]	NS	-	-	-	-	-	-	-
He 2019 [26]	NS	-	-	NS	-	-	60° NS 240° HT	60° BPTB 240° BPTB
	HT			-	-	-	-	
	NS trend to favor HT (p=0,08)			NS	-	-	60° NS 240° -	60° NS 240° -
Chen 2019 [27]	-	NS	NS	NS	-	-	-	-
Zhao 2020 [28]	NS	NS trend to favor BPTB (p=0,06)	NS	NS	-	-	-	-
Chen 2020 [29]	NS	NS trend to favor BPTB (p=0,09)	NS	NS	NS	NS	-	-
Zhou 2020 [30]	-	-	-	-	-	-	-	-

**Table 3:** Static stability outcomes NS: non-significant difference, HT: in favor of hamstrings autograft, BPTB: in favor of bone patella tendon bone autograft

Meta-analysis	SIDE TO SIDE DIFFERENCES	LACHMAN	PIVOT
Yunes 2001 [5]	NS trend to favor BPTB (p=0,06)	NS	BPTB (p=0.05)
Freedman 2003 [6]	BPTB	-	NS
Forster 2005 [7]	Data not combined or NS	NS	NS
Goldblatt 2005 [8]	BPTB	NS trend to favor BPTB (p=0,06)	NS trend to favor BPTB (p=0,09)
Prodromos 2005 [9]	4HT	-	-
Biau 2006 [10]	BPTB	BPTB	NS
Biau 2007 [11]	-	-	-
Poolman 2007 [12]	-	BPTB	-
Biau 2009 [13]	-	NS	BPTB
Magnussen 2010 [14]	NS	-	-
Mohtadi 2011 [15]	BPTB	BPTB	BPTB
Li 2011 [16]	BPTB	NS	BPTB
Xergia 2011 [17]	-	-	-
Li 2012 [18]	-	NS	BPTB
Ardern 2014 [19]	-	-	-
Xie 2014 [20]	NS	NS	NS
Xie 2015 [21]	NS trend to favor BPTB (p=0,06)	NS	BPTB
Gabler 2016 [22]	-	-	-
Chee 2016 [23]	NS	NS	NS
Samuelsen 2017 [24]	NS	NS	NS
Tan 2018 [25]	NS	NS	NS
He 2019 [26]	BPTB	-	BPTB
	NS	NS	NS
	NS	NS	NS
Chen 2019 [27]	NS	NS trend to favor BPTB (p=0,08)	NS trend to favor BPTB (p=0,06)
Zhao 2020 [28]	NS	NS	NS trend to favor BPTB (p=0,09)
Chen 2020 [29]	NS	NS	NS trend to favor BPTB (p=0,07)
Zhou 2020 [30]	-	-	-

**Table 4:** Postoperative complications KP: kneeling pain, AKP: anterior knee pain, Ext.loss: extension loss, Flex.loss: flexion loss, GF: graft fracture, CACLR: contralateral ACL rupture, OA: osteoarthritis, NS: nonsignificant difference, HT: in favor of hamstrings autograft, BPTB: in favor of bone patella tendon bone autograft

Meta-analysis	KP	AKP	Ext.loss	Flex.loss	GF	Infection rate	CACLR	OA
Yunes 2001 [5]	-	-	Data not combined	NS	NS	-	-	-
Freedman 2003 [6]	-	HT	NS	Data not combined	BPTB	NS	-	-
Forster 2005 [7]	-	NS trend to favor HT (p=0,09)	HT	Data not combined	NS	-	-	-
Goldblatt 2005 [8]	HT	NS trend to favor HT (p=0,12)	NS trend to favor HT (p=0,06)	BPTB	NS	-	-	-
Prodromos 2005 [9]	-	-	-	-	-	-	-	-
Biau 2006 [10]	HT	HT	HT	-	NS	-	-	-
Biau 2007 [11]	-	-	-	-	-	-	-	-
Poolman 2007 [12]	-	HT	-	-	-	-	-	-
Biau 2009 [13]	-	-	-	-	-	-	-	-
Magnussen 2010 [14]	-	-	-	-	NS	-	-	-
Mohtadi 2011 [15]	HT	HT	HT	NS	NS	-	-	-
Li 2011 [16]	HT	HT	HT	-	NS	-	-	-
Xergia 2011 [17]	-	-	-	-	-	-	-	-
Li 2012 [18]	HT	HT	NS trend to favor HT (p=0,05)	NS	NS	NS	-	-
Ardern 2014 [19]	-	-	-	-	-	-	-	-
Xie 2014 [20]	HT	HT	NS trend to favor HT (p=0,09)	NS	NS	-	-	HT
Xie 2015 [21]	HT	HT	NS trend to favor HT (p=0,06)	NS	NS	-	-	-
Gabler 2016 [22]	-	-	-	-	-	-	-	-
Chee 2016 [23]	HT	HT	HT	-	NS	-	-	-
Samuelsen 2017 [24]	-	-	-	-	BPTB	-	-	-
Tan 2018 [25]	NS	NS	-	-	NS	-	-	-
He 2019 [26]	HT	HT	NS	-	NS	NS	NS	-
	HT	NS	-	-	NS	NS	NS	-
	NS	HT	NS	-	NS	NS	NS	-
Chen 2019 [27]	-	-	-	-	BPTB	-	-	-
Zhao 2020 [28]	HT	HT	HT	NS	BPTB	-	-	NS
Chen 2020 [29]	NS trend to favor BPTB (p=0,07)	HT	NS	NS	NS	-	-	NS
Zhou 2020 [30]	-	-	-	-	-	-	HT	-

1. IKDC score: Overall, 13 meta-analyses used IKDC scores to compare the two grafts [6-8,11,15,18,20-21,23,25-26,28-29], of which 10 found no statistically significant difference between the two groups [8,11,15,18,20-21,23,25,28-29] and two could not combine data due to significant heterogeneity [6-7]. Only He et al. reported a statistically significant difference favoring the HT group in the mid-term period and a trend towards favoring the HT group in the long-term period but no difference was found in the short-term outcomes [26].

2. Lysholm knee score: Nine studies reported Lysholm knee scores [6-8,15,21,23,27-29] and six of them did not manage to find any statistically significant difference between the two grafts [15,21,23,27-29]. Zhao et al. and Chen et al. found only a trend in favor of the BPTB group regarding Lysholm scores [28-29]. Freedman et al. and Foster et al. did not manage to combine data due to significant heterogeneity between the studies [6-7] and Goldblatt et al. could only perform graphical analysis with no apparent differences between the two grafts [8].

3. RIAL: Thirteen of 26 meta-analyses presented results on this topic [5-8,11,15,19-21,23,27-29] and only three managed to find a statistically significant difference favoring the BPTB [5,19,21].

4. Tegner knee score: Seven meta-analyses reported on the Tegner knee score outcome and none concluded in a statistically significant difference between the two grafts [8,15,23,26-29]. Freedman et al. could not combine data due to significant heterogeneity [6] and Goldblatt et al. demonstrated only a graphical analysis with no apparent differences [8].

5. Side-to-side differences (SSD): Eighteen meta-analyses presented data on side-to-side differences by instrumented laxity [5-10,14-16,20-21,23-29], and 10 of them found no statistically significant difference. Yunes et al. and Xie et al. found a trend toward favoring the BPTB graft though this did not reach significance [5,21]. Forster et al. could not combine data due to lack of information but reported that even if data were combined, there was no significant difference between the groups [7]. Five meta-analyses found a statistically significant difference favoring the BPTB autograft [6,8,10,15-16] while He et al. found that short-term outcomes favored the BPTB group but no differences were found in mid- and long-term evaluations [26]. Only Prodromos et al. found a higher stability rate in favor of the HT graft but only when BPTB was compared to the four-strand technique [9].

6. Lachman test: Eighteen meta-analyses demonstrated the data of the Lachman test [5,7-8,10,12,13,15-16,18,20-21,23-29], and 15 of them found no statistically significant difference between the two grafts [5,7-8,13,16,18,20-21,23-29]. He et al. did not find any statistically significant difference between the BPTB and HT autografts in the mid- and long-term periods and short-term outcomes weren’t available [26]. Goldblatt et al. and Chen et al. reported a trend toward favoring BPTB autograft [8,27]. Biau et al., Poolman et al., and Mohtadi et al. found that BPTB had significantly lower rates for positive Lachman compared to HT [10,12,15].

7. Pivot-shift test: Pivot shift was reported in 18 of the meta-analyses [5-8,10,13,15-16,18,20-21,23-29]. Eleven found no statistically significant difference between the two grafts [6-8,10,20,23-25,27-29] while four of them found a trend toward favoring the BPTB graft [8,27-29]. Six meta-analyses found a significant difference in favor of BPTB [5,13,15-16,18,21] while He et al. found that short-term outcomes favored the BPTB group but no difference was found in mid- and long-term evaluations [26].

8. Kneeling pain: Twelve studies reported on kneeling pain [8,10,15-16,18,20-21,23,25-26,28-29], and nine of them found that the HT graft was superior to BPTB in this outcome [8,10,15-16,18,20-21,23,28]. Tan et al. reported no significant difference between the two grafts, however, they only reported on the female population [25]. Chen et al. also reported no statistically significant difference but found a trend to favor the HT graft [29]. He et al. found the HT autograft to be superior to the BPTB autograft in short- and mid-term outcomes but no difference was reported between them in long-term outcomes [26].

9. Anterior knee pain: Fifteen of the meta-analyses reported on anterior knee pain [6-8,10,12,15-16,18,20-21,23,25-26,28-29], with 11 of them reporting outcomes in favor of the HT autograft [6,10,12,15-16,18,20-21,23,28-29]. Only three found no statistically important difference between the two grafts [7,8,25], with two of them reporting a trend toward favoring the HT graft [7-8] and the third one [25] reporting only on the female population. He et al. favored the HT autograft over the BPTB autograft in short- and long-term outcomes but found no difference between them in mid-term outcomes [26].

10. Extension loss: Extension loss was reported in 14 of the meta-analyses [5-8,10,15-16,18,20-21,23,26,28-29] with six of them reporting more extension deficits in the BPTB group [7,10,15-16,23,28]. Seven did not find any statistically significant difference between the two groups [6,8,18,20-21,26,29] but four of them reported a trend toward favoring the HT graft [8,18,20-21]. He et al. did not find any statistically significant difference between the two groups in the short- and long-term evaluation but mid-term outcomes weren’t available [26]. Yunes et al. were unable to pool data because of significant heterogeneity among study results [5].

11. Flexion loss: Ten meta-analyses reported on flexion loss [5-8,15,18,20-21,28-29] with seven of them reporting no significant difference between the two grafts [5,15,18,20-21,28-29]. Freedman et al. and Forster et al. could not reach an outcome since the data pool contained too much heterogeneity to allow for meaningful analysis [6-7]. Goldblatt et al. was the only meta-analysis that found a statistically significant difference in favor of the BPTB graft [8].

12. Graft failure: Eighteen meta-analyses reported data on graft failure [5-8,10,14-16,18,20-21,23-29], and 14 of them found no statistically significant difference between the two grafts [5,7-8,10,14-16,18,20-21,23,25-26,29]. The other four meta-analyses reported that graft failure rates were higher in the HT group [6,24,27-28].

13. Progression to osteoarthritis: Only three meta-analyses reported outcomes on osteoarthritis since it is considered a long-term complication [20,28-29]. Zhao et al. and Chen et al. did not find any statistically significant difference between the two graft types [28-29] while Xie et al. showed that the incidence of OA was significantly higher in the BPTB group compared to the HT group [20].

14. Cincinnati Knee Rating System: Outcomes for the Cincinnatti Knee Rating System were found only in three meta-analyses, and all reported no statistically significant difference between the two grafts [7,15,29].

15. Single leg hop test: The only two meta-analyses that reported on the single-leg hop test were Mohtadi et al. and Chen et al., and both found no statistically significant difference among the two groups [15,29].

16. Extension strength: Five meta-analyses pooled data to evaluate extension strength between the two grafts [7-8,15,17,26]. Forster et al. and Mohtadi et al. found no differences between the BPTB and HT grafts in terms of peak quadriceps torque at 60° speed [7,15]. Mohtadi et al. reported the same results at 180° [15]. He et al. found no statistically significant difference at 60° in the short- and long-term evaluations but found HT to be superior to the BPTB graft at the speed of 240°/s. in short-term outcomes [26]. Xergia et al. showed that the difference between the BPTB and HT groups for extensor muscle strength was nearly 10% at the speed of 60°/s and 180°/s in favor of the HT autograft [17]. Goldblatt et al. could not perform an analysis of extension strength because of wide variability in reporting among studies [8].

17. Flexion strength: Five studies reported outcomes in flexion strength deficits, with four of them noting statistically significant differences in favor of the BPTB autograft [7,15,17,26]. More specifically, Forster et al. and Mohtadi et al. both found BPTB to be superior to the HT graft in terms of flexion strength at 60° and 180° [7,15]. He et al. favored the BPTB graft for both 60° and 240° speeds in short-term measurements but no difference was found in the long-term [26]. Xergia et al. showed that the difference between the BPTB and HT group for flexor muscle strength was as much as 20% at the speed of 180°/s [17]. Goldblatt et al. could not perform an analysis for flexion strength because of the wide variability in reporting among studies [8].

18. Infection rate: All three meta-analyses that pooled data to compare the infection rate between the two grafts found no statistically significant difference between them [6,18,26]. Specifically, He et al. found no difference in none of the three, short, mid and long-term periods [26].

19. Contralateral ACL rupture (CACLR): Only two recent meta-analyses presented data on CACLR [26,30]. He et al. found no significant difference between the two grafts in terms of CACLR in all three follow-up periods in contrast to Zhou et al. who reported lower rates of CACLR for the HT autograft [26,30].

Subgroup Analyses

A subgroup analysis was conducted by nine meta-analyses according to the type of study design (i.e. RCT versus non-RCT studies), the number of strands of HT autograft, and the fixation method (Table 5).

**Table 5:** Subgroup analyses AKP: anterior knee pain, SSD: side-to-side differences, Ext.loss: extension loss, Flex.loss: flexion loss, Flex.str: flexion strength, Ext.str: Extension strength, GF: graft fracture, RIAL: return to preinjury activity level, NS: nonsignificant difference, HT: in favor of hamstrings autograft, BPTB: in favor of bone patella tendon bone autograft

Meta-analysis	Overall analysis	Subgroup analysis (for RCTs)	Subgroup analysis (for non-RCTs)	Subgroup analysis (for 4HT)	Subgroup analysis (for less than 4 strands)	Subgroup analysis (for endobutton)	Subgroup analysis (for older fixation techniques e.g. Screws)
Biau 2006 [10]	Pivot: NS AKP: HT SSD: BPTB Lachman: BPTB	Pivot: NS AKP: HT	Pivot: NS AKP: HT	Pivot: NS SSD: NS Lachman: NS	Pivot: NS (p=0.05)		
Poolman 2007 [12]	Lachman: BPTB					Lachman: NS	Lachman: BPTB
Mohtadi 2011 [15]	IKDC: NS Tegner: NS Lysholm: NS SSD: BPTB Lachman: BPTB Pivot: BPTB Ext. loss: HT Flex. Loss: NS Flex.str.60°:BPTB Ext.str.60°: NS AKP: HT GF: NS	IKDC: NS Tegner: NS Lysholm: NS SSD: BPTB (0.05) Lachman: NS Pivot: BPTB Ext.loss: HT Flex. Loss: BPTB Flex. str.60°: NS Ext.str.60°: NS AKP: NS GF: NS	IKDC: NS Lachman: BPTB Pivot: NS Ext. Loss: NS Flex. Loss: NS Ext.str.60°: NS AKP: HT GF: NS	IKDC: NS SSD: BPTB Lachman: NS Pivot: BPTB Ext. Loss: HT Flex. Loss: NS Flex.str.60°: BPTB Ext.str.60°: NS AKP: HT GF: NS	IKDC: NS SSD: BPTB Lachman: NS Pivot: BPTB Ext. loss: NS Flex. loss: NS Flex.str.60°: NS Ext.str. 60°: NS AKP: NS GF: NS	IKDC: NS SSD: NS Lachman: NS PIivot: NS (0,06) Ext.loss: HT Flex. Loss: NS Flex.str 60°: NS Ext.str 60°: NS AKP: NS GF: NS	IKDC: NS SSD: BPTB Lachman: NS Pivot: NS (0,09) Ext.loss: HT Flex. Loss: NS Flex.str.60°: BPTB Ext.str. 60°: NS AKP: NS GF: NS
Li 2011 [16]	Pivot: BPTB	Pivot: BPTB	Pivot: NS	PIivot: NS	Pivot: BPTB	PIivot: NS	Pivot: NS (trend to favor BPTB p=0.09)
Ardern 2014 [19]	RIAL: BPTB	RIAL: NS					
Xie 2014 [20]	SSD: NS Lachman: NS Pivot: NS IKDC: NS GF: NS	SSD: NS Lachman: NS Pivot: NS IKDC: NS GF: NS					
Xie 2015 [21]	SSD: NS Lachman: NS Pivot: BPTB IKDC: NS GF: NS	SSD NS (trend to favor BPTB p=0.05) Lachman: NS Pivot: BPTB IKDC: NS GF: NS	SSD: NS Lachman: NS Pivot: NS IKDC: NS GF: NS				
Samuelsen 2016 [24]	SSD NS Lachman NS Pivot NS GF BPTB	SSD: BPTB Lachman: NS Pivot: NS GF: BPTB	SSD: NS Lachman: NS Pivot: NS GF: BPTB				
Chen 2019 [27]	Lysholm: NS Tegner: NS SSD: NS Lachman: NS Pivot: NS GF: BPTB	Lysholm: NS Tegner: NS SSD: NS Lachman: NS Pivot: NS GF: NS (trend to favor BPTB p=0.09)	Lysholm: NS Tegner: NS SSD: NS Lachman: NS Pivot: NS GF: NS				

Chen et al. performed subgroup analysis based on the type of study design (RCTs or non-RCTs) for the outcomes of Lysholm, Tegner, SSD, Lachman, pivot, and graft failure. No differences were found in the overall analysis with the exception of graft failure, where a trend was found in favor of BPTB in the RCTs subgroup analysis. [27]

Samuelsen et al., who used only the four-strand HT autograft in their meta-analysis compared to the BPTB autograft, also conducted subgroup analyses in regards to the type of study design for the outcomes of SSD, Lachman, pivot, and graft failure [24]. The difference was only found for the SSD in favor of the BPTB autograft in the RCTs' subgroup analysis. All other outcomes remained consistent with the overall analysis.

Xie et al., who also compared BPTB to only four-strand graft, found no differences for Lachman, IKDC, and graft failure outcomes neither in the overall analysis nor their subgroup analyses for RCTs and non-RCTs [21]. They did, however, find a difference in their subgroup analysis of non-RCTs (PCS) for pivot test, which was found to be of no statistically significant difference between the two grafts, in contrast to the overall analysis where they favored BPTB graft. The side-to-side difference was found similar to all three analyses (overall and subgroups) between the two grafts but with a trend towards favoring BPTB graft (p=0.05) in the RCTs subgroup analysis. Additionally, Xie et al., using only 4HT in their meta-analysis, found no differences in the overall analysis and in the subgroup analysis of RCTs for SSD, Lachman, pivot, IKDC, and graft failure outcomes [20].

Ardern et al. [19] noted that when data from four randomized studies reporting on RIAL were combined, there was no statistically significant difference between the two grafts in contrast to the overall analysis, where RIAL was found in favor of BPTB autograft. A fifth randomized study included in their meta-analysis by Ibrahim et al. [32] that reported on RIAL, did not present separate data for graft type but stated that there was no statistical difference in the rate of return to preinjury level of activity between BPTB and HT grafts.

Li et al. conducted subgroup analyses according to the type of the study design as well as the number of strands for HT graft and the fixation method. They found the pivot test to be in favor of the BPTB graft in the subgroup analyses of RCTs and less-than-four-strand hamstrings graft, as was reported to the overall analysis. However, no difference was found between the two grafts when BPTB was compared only to the four-strand HT graft. No difference was also found when the endobutton was used as a fixation method and only a trend toward favoring BPTB when older fixation methods were used [16].

Mohtadi et al. also conducted several subgroup analyses regarding the type of the study design, the number of the strands of hamstrings autograft, and the fixation technique [15]. Outcomes were found for IKDC, Tegner, Lysholm, SSD, Lachman, pivot, AKP, extension and flexion loss, extension and flexion strength at 60°, and graft failure. Differences to the overall analysis were found regarding 1. SSD, in the subgroup analysis using the endobutton, where no statistically significant difference between the two groups was noted, 2. Lachman test, at the subgroup analyses of RCTs, HT graft with four strands, HT graft with < 4 strands, HT femoral fixation with the endobutton, and HT femoral fixation with screw, where no statistically significant difference between the two grafts was found, 3. pivot test, at the subgroup analyses of quasi-RCTs, HT femoral fixation with the endobutton and HT femoral fixation with screw, where there was also no statistically significant difference between the two grafts, 4. extension loss, at the subgroup analyses of quasi-RCTs and HT graft with < 4 strands, where there was no difference between the BPTB and HT grafts, 5. flexion loss, at the subgroup analysis of RCTs, favoring BPTB compared to HT, 6. flexion strength at 60°, at the subgroup analyses of RCTs, HT graft with < 4 strands and HT femoral fixation with the endobutton, where no statistically significant difference was found, and 7. AKP, at the subgroup analyses of RCTs, HT graft with < 4 strands, HT femoral fixation with endobutton, and HT femoral fixation with screw, where no statistically significant difference was found too.

Poolman et al. conducted subgroup analysis regarding the fixation method and concluded that using endobutton as a fixation technique resulted in equal rates in the Lachman test between the two groups while in their overall analysis, Lachman outcomes were in favor of the BPTB graft [12].

Biau et al. performed subgroup analyses regarding the type of the study design and the number of the strands of hamstrings autograft and found no differences compared to the overall analysis for pivot and AKP outcomes [10]. Nevertheless, they reported that when they compared only the four-strands HT graft to BPTB graft, there were no statistically significant differences between the two groups in SSD and Lachman outcomes in contrast to the overall analysis that favored BPTB graft.

Discussion

The purpose of this study was to strengthen the documentation of autograft choice between BPTB and HT used for ACL rupture.

The IKDC, Lysholm, and Tegner scores can provide an overall evaluation of postoperative ACL reconstruction outcomes. All 26 meta-analyses reported no statistically significant differences in these three outcomes, neither in their overall analysis nor in the subgroup analyses when performed. This is a strong indicator that neither graft is superior with regards to functional assessment and patients‘ reported outcomes.

The primary purpose of ACL reconstruction is to help patients return to their pre-injury activity levels. The majority of the meta-analyses found no significant difference between the two groups in returning to the pre-injury activity levels while Ardern et al. found a statistically significant difference in favor of BPTB group, which was equalized when only RCTs were included [19]. They also examined the importance of other factors in returning to the pre-injury activity level such as gender, age, improved physical functioning, and psychological factors. They resulted that younger age, male gender, playing elite sports, and having a positive psychological response were contextual factors that favored returning to the pre-injury level sport and while they showed that people who received BPTB autografts had greater odds of returning to their preinjury level sport, people who received hamstring tendon autografts had greater odds of returning to competitive level sport. All these factors have to be taken into consideration in future studies.

Instrumented laxity is a very important evaluation tool of the successful outcome after an ACL reconstruction. From our findings, we can see that a side-to-side difference was only found in favor of the BPTB autograft when compared to the less than four-strand autograft. This difference was of no significance between the two groups when compared to four strands only, like in the subgroup analysis of Biau et al., or to HT femoral fixation with the extra-cortical button, like in the subgroup analysis of Mohtadi et al. [10,15]. This indicates the superiority of the four-strand autograft compared to the fewer strands hamstring autograft and the superiority of the fixation with the extra-cortical button. Only Prodromos et al. found a higher stability rate in favor of HT graft but only when BPTB was compared to the four-strand technique [9]. They were also the first study to analyze ACL reconstruction stability results as a function of fixation type for either HT or BPTB grafts and show that four-strand HT autograft stability rates are fixation dependent, with extra-cortical button combined with second-generation tibial fixation producing consistently high-stability rates.

The same results were demonstrated in the meta-analysis by Poolman et al. and Biauet al., who found Lachman to be in favor of the BPTB group in their overall analyses, but when restricted to the four-strand HT autograft with femoral fixation of endobutton, the two autografts seemed to be equal regarding this outcome [10,12]. Mohtadi et al. while they reported significantly lower rates for positive Lachman for the BPTB group compared to the HT group, they did not find the same results in any of the subgroup analysis they conducted, except the subgroup analysis of quasi-RCTs, and this is strong evidence of the impact non-RCTs have on the general results [15].

The pivot shift is commonly used to assess the combined tibiofemoral internal rotation and anterior tibial translation, which, from our review, seemed to be in favor of the BPTB group in six meta-analyses [5,13,15-16,18,21]. It is important to note that four of these meta-analyses compared the BPTB autograft to the multi-strands HT autograft [5,15-16,18]. From the other two that compared the BPTB autograft to four-strand HT autograft, one was limited to RCTs [13] and the other favored BPTB in the overall analysis as well as in the subgroup analysis of RCTs, however, found no difference between the two groups, in the subgroup analysis of non-RCTs, indicating once more the impact non-RCTs have to the overall analysis [21]. Every other meta-analysis that compared the BPTB autograft to the four-strand autograft only and limited its research to RCTs found no difference between the two grafts with the exception of He et al., who, while supporting this outcome of lower rates of positive pivot test for BPTB patients in the short-term follow-up, could not find the same results in long-term follow-up [26]. Any difference between the two grafts seemed to equalize with time and shows the need for more future studies to make a conclusion regarding the long-term functional outcome differences between the two graft types.

As shown in Table 4, kneeling pain and anterior knee pain were quite common after ACL reconstruction with BPTB autograft, and this is supported strongly by numerous studies throughout the years. The reduction of donor-site morbidity by using HT autografts is the main concern of patients and surgeons. A study by Mastrokalos et al. showed that a high rate of patients had pain, loss of sensitivity, or both at the donor site after ACL reconstruction with a BPTB graft, with most experiencing these symptoms almost two or three years after the operation [33].

Extension and flexion loss is determined as the loss of extension or flexion in the operated knee in comparison with the contralateral healthy knee. From our review, it is shown that the outcome of extension loss may also be interconnected with the number of strands of the HT autograft and the type of study design. More specifically, while Mohtadi et al. found a statistically significant difference in favor of the HT group regarding extension loss, in their overall analysis, this outcome changed to no significant difference between the two groups, when BPTB was compared only to the less than four-strand HT autografts [15]. This may contradict the findings of Xie et al. who reported no significant difference in their meta-analyses between the BPTB and the four-strand HT autograft regarding the extension loss, but we have to take into account that their meta-analyses weren’t limited in RCTs only [20-21]. In addition, He et al. and Chen et al., who compared the BPTB autograft to only the four-strand HT autograft and used only RCTs in their meta-analyses, found no difference between them regarding extension loss [26,29]. Data on flexion loss is more limited, however, there is evidence that there is no difference between the two groups.

Graft failure is the greatest fear of every patient and the main concern of every surgeon, and it seems to be less common in the BPTB autograft group. This may be due to the fact that the time required for the BPTB graft to incorporate and heal is shorter than the time the HT graft needs.

Regarding the progression of osteoarthritis, while Xie et al. showed that the BPTB group had higher rates of osteoarthritis, Zhao et al. and Chen et al. in two more recent meta-analyses including only RCTs, reported no difference between the two groups [20,28-29]. Therefore, this issue remains controversial and there is a need for more recent high-quality RCTs to enhance the latest evidence for clinical decision-making.

Flexion and extension strength are two outcomes that seem to be affected by the donor site, but we did not find this topic frequently examined and more studies are needed to reach safer results.

Regarding CACL rupture, in our review, only two meta-analyses reported data, with one of them being in favor of HT autografts and the other one not finding any differences between the two groups. However, previous studies indicated that patients undergoing primary ACL reconstruction were more likely to experience CACL rupture with BPTB versus HT autograft [34-37]. One hypothesis may be that patients who received BPTB autograft return to more competitive sports and may protect the operated knee out of fear of reinjury, leaving the CACL unguarded. Generally, the reported incidence of contralateral ACL (CACL) injury varies between 0.6% and 22.7%, and this may be attributed to different follow-up periods in individual trials [38]. More research on this topic is needed, with high-quality RCTs of longer follow-up periods. The same necessity is exhibited by our review for the outcomes of Cincinnati, single-leg hop test, and infection rate.

## Conclusions

Numerous meta-analyses have focused on comparing BPTB and HT autografts in order to present which is optimal for ACL reconstruction. Although some outcomes are consistent throughout studies, like the superiority of the HT autograft over BPTB in regards to kneeling pain and anterior knee pain, others seem to be affected factors like the number of strands of the hamstrings autograft, the fixation technique, and the type of study design. Similarly, several other factors, such as younger age, male gender, playing elite sports, and having a positive psychological response can affect postoperative outcomes. While some researchers consider BPTB to be the gold standard, a four-strand HT autograft with extra-cortical button fixation seems to be a favorable choice. In any case, autograft selection should be individualized according to every patient’s needs, and more research on this topic is required. High-quality RCTs with long-term follow-up are one of the best ways to evaluate a surgical technique due to their potential to limit all sorts of biases.
